# CAR T cells as novel therapeutic strategy for multiple sclerosis and other neuroimmune disorders

**DOI:** 10.1186/s12974-025-03668-0

**Published:** 2025-12-30

**Authors:** Sara Samadzadeh, Natalia Szejko, Yara Hamadah, Wan Ying Tan, Sanja Gluscevic, Vinícius Boldrini, Vito A. G. Ricigliano, Hans-Peter Hartung, Xavier Montalban, ECF Young Investigators/Fellows Initiative

**Affiliations:** 1 Experimental and Clinical Research Center, Charité-Universitätsmedizin Berlin, Corporate Member of Freie Universität Berlin and Humboldt- Universität zu Berlin, Berlin, 12203 Germany; 2 Institute of Regional Health Research, Institute of Molecular Medicine, University of Southern Denmark, Odense, 5230 Denmark; 3 The Center for Neurological Research, Department of Neurology, Naestved- Slagelse-Ringsted Hospitals, Slagelse, 4200 Denmark; 4 Department of Bioethics, Medical University of Warsaw, Warsaw, Poland; 5 Department of Experimental and Clinical Pharmacology, Center for Preclinical Research and Technology CEPT, Medical University of Warsaw, Warsaw, Poland; 6 Clinic of Psychiatry, Social Psychiatry and Psychotherapy, Hannover Medical School, Hannover, Germany; 7 Department of Internal Medicine, University of Connecticut School of Medicine, Farmington, CT USA; 8 Department of Hematology & Oncology, Neag Comprehensive Cancer Center, University of Connecticut School of Medicine, Farmington, CT USA; 9 Neurology Clinic, Clinical Centre of Montenegro, Podgorica, 81000 Montenegro; 10 Brazilian Institute of Neuroscience and Neurotechnology (BRAINN), Department of Neurology, University of Campinas (UNICAMP), Campinas, São Paulo, Brazil; 11 Neurology Unit, Paris Saclay Hospital, Orsay, France; 12 Université Paris-Saclay, UNIACT, Neurospin - INSERM UMR 1129, Gif-sur- Yvette, France; 13 Brain and Mind Center, University of Sydney, Sydney, 2050 Australia; 14 Department of Neurology, Palacky University Olomouc, Olomouc, 779 00 Czech Republic; 15 Department of Neurology, Medical Faculty, Heinrich-Heine University, Düsseldorf, 40225 Germany; 16 Multiple Sclerosis Centre of Catalonia (CEMCAT) and Department of Neurology, Hospital UniversitaryVall d´Hebron, Universitat Autonoma de Barcelona and Universitat de Vic/Central de Catalunya (UVic/UCC), Barcelona, Spain; 17 Department of Child Psychiatry, Babinski Hospital, Lodz, Poland

**Keywords:** Chimeric antigen receptor t cells, Multiple sclerosis, Autoimmune neurology, Autoimmune disorders, B cells, Neuroinflammation, Immunotherapy

## Abstract

Chimeric antigen receptor (CAR) T-cell therapy is rapidly emerging as a transformative approach for treating multiple sclerosis (MS) and other neuroimmune disorders such as neuromyelitis optica spectrum disorder (NMOSD), myelin oligodendrocyte glycoprotein antibody-associated disease (MOGAD), and myasthenia gravis (MG), alongside several other rare neuroimmunological conditions currently being evaluated in compassionate-use or early-phase studies. These conditions are driven in part by autoreactive B cells that sustain chronic inflammation and progressive tissue damage. While current immunomodulatory therapies have improved clinical outcomes, they often require lifelong administration and fail to effectively eliminate compartmentalized inflammation within the central nervous system. Recent advances in CD19- and BCMA-directed CAR T-cell therapy, initially developed for hematologic malignancies, demonstrate the potential to achieve targeted, durable B-cell depletion and immune reprogramming in autoimmune diseases. Preclinical models and early-phase clinical trials have shown promising efficacy, including reduced relapse rates, stabilization of disability progression, and decreased autoantibody levels, alongside a favorable safety profile with lower rates of high-grade cytokine release syndrome (CRS) and immune effector cell-associated neurotoxicity syndrome (ICANS) compared to oncologic applications. This review synthesizes the current evidence supporting the use of CAR T-cell therapy in neuroinflammatory diseases and explores its potential to redefine treatment paradigms by shifting from chronic immunosuppression to long-term immune tolerance, creating a favorable environment for repair mechanisms. Realizing the full therapeutic promise of CAR T-cells in autoimmune neurology will require sustained research in heterogeneous populations and across disease spectrums.

## Introduction: unmet needs in MS management and the potential role of CAR T-cell therapy

Multiple sclerosis (MS) more frequently presents as relapsing disease (RMS), characterized by periods of acute relapses interrupting a relatively stable disease course. During these periods, inflammatory lesions surpass the clinical threshold, leading to symptoms that contribute to disability accumulation (relapse-associated worsening, or RAW). In addition to focal events, many people with MS (PwMS) show a more insidious, progressive accumulation of disability, occurring independently of relapses, defined as progression independent of relapse activity (PIRA) [1].

Episodic relapses and RAW are caused by acute neuroinflammation, triggered by peripheral immune activity. In contrast, chronic neuroinflammation, which takes place within the central nervous system (CNS) and is characterized by chronic active lesions and progressive brain atrophy, is the leading mechanism of PIRA. Both RAW and PIRA contribute to overall disability accrual, highlighting the need for treatment strategies that address both mechanisms [2].

B cells play a crucial role in MS pathology through their involvement in both peripheral and CNS inflammation. In the periphery, autoreactive B cells act as antigen-presenting cells (APCs) to helper (CD4^+^) T-cells, initiating inflammatory cascades. Regulatory B cells (Bregs) typically suppress effector T-cell (Teff) activity; however, in MS, Bregs may be dysfunctional, disrupting immune regulation and leading to heightened activation of autoreactive B cells [3–5]. Once B cells enter the CNS, they activate astrocytes and microglia, releasing cytokines such as TNF-α, IL-6, and GM-CSF, which amplify neuroinflammation. Some B cells also differentiate into plasmablasts and plasma cells, producing IgG autoantibodies that exacerbate neural damage. Additionally, ectopic germinal centers may form in the meninges, fostering a localized microenvironment that sustains chronic B- and T-cell activation within the CNS [6–8].

Anti-CD20 therapies target B cells and have shown effectiveness in reducing disability progression in both RMS and primary progressive MS (PPMS). By depleting CD20-expressing B cells, these therapies reduce relapse rates and slow peripheral inflammation-driven progression. Studies have demonstrated that anti-CD20 treatment significantly delays 24-week confirmed disability progression (CDP) in RMS patients compared to control groups. Similar reductions in CDP have been observed in PPMS over extended periods [9, 10]. Nonetheless, anti-CD20 therapies are limited in addressing CNS-compartmentalized inflammation, due to their inability to penetrate the blood-brain barrier (BBB) [11, 12], making them incompletely effective against chronic CNS inflammation [13].

In patients treated with anti-CD20 therapy, PIRA events account for the majority of disability accumulation. Approximately 89.1% of 24-week composite disability accumulation (CDA) events are attributed to PIRA, while only 12.4% result from RAW, indicating that relapse-independent mechanisms drive long-term disability, even with effective relapse suppression [13]. Additionally, although higher exposure to anti-CD20 therapy was previously associated with reduced CDP risk [14] and suggested a dose-dependent relationship across different body mass index (BMI) categories [15], recent evidence from the Phase 3b GAVOTTE (NCT04548999) and MUSETTE (NCT04544436) trials showed that higher-dose ocrelizumab regimens do not provide additional clinical benefit over the approved 600 mg dose. Both studies reported comparable outcomes in cognition, walking function, and dexterity, with similar safety profiles, reinforcing the efficacy plateau of current anti-CD20 strategies and supporting the need for next-generation, deeper immune-modulating approaches [16].

Moreover, prolonged B-cell depletion with anti-CD20 raises concerns regarding infections and hypogammaglobulinemia. Long-term anti-CD20 therapy (≥ 3 years) has been linked to increased incidences of serious infections and low immunoglobulin levels, underscoring the need for careful monitoring and individualized risk assessment in long-term management [17, 18].

The limited impact of anti-CD20 molecules on long-term disability mitigation underscores a critical therapeutic gap, particularly in progressive phenotypes driven by CNS-compartmentalized inflammatory processes refractory to peripherally restricted monoclonal antibodies [19]. This pathophysiological dichotomy,, acute versus chronic neuroinflammation,, necessitates therapeutic strategies capable of targeting both dynamic overt disease activity and persistent CNS-resident pathology. Emerging approaches include CNS-penetrant Bruton’s tyrosine kinase inhibitors (BTKIs), with six agents under investigation in phase II/III trials for relapsing and progressive MS [20]. Parallel efforts are evaluating next-generation CD20 monoclonal antibodies engineered to cross the BBB via receptor-mediated transcytosis, enabling direct CNS-targeted B-cell depletion. A first-in-human study of such an agent is underway (NCT05704361).In this scenario, emerging Chimeric Antigen Receptor (CAR) T-cell therapies targeting CD19 or B-cell maturation antigen (BCMA) may offer deeper and more comprehensive immune modulation than conventional B-cell depletion. Unlike anti-CD20 monoclonal antibodies, CD19-directed CAR T-cells have been detected in CSF after infusion, indicating potential access to CNS-compartmentalized B-cell niches. By targeting a broader spectrum of B-cell populations, including naïve, memory, plasmablast, and CD19⁺ early plasma cells, CAR T-cell approaches may achieve more profound and longer-lasting B-cell depletion. Early autoimmune CAR T-cell studies suggest depletion lasting 6–12 months, though durability varies across diseases and products [21–23].

The concept of an “immune reset” in CAR T-cell therapy refers to the temporary ablation of pathogenic B-cell lineages followed by repopulation with immunologically naïve or less autoreactive clones, a phenomenon supported by observations in systemic lupus erythematosus (SLE) and myasthenia gravis (MG), where clinical remission persisted despite peripheral B-cell reconstitution. However, true long-term immune tolerance has not yet been demonstrated, and current evidence is limited to small early-phase cohorts with short follow-up [24].

Regarding IgG-mediated pathology in MS, the reference is to intrathecal IgG production, complement deposition at active lesion edges, and the beneficial effect of plasma exchange during fulminant relapses, suggesting an antibody-driven component despite the absence of a single known target antigen. Nonetheless, CAR T-cell effects on intrathecal IgG in MS remain preliminary and require confirmation in larger clinical studies [25].

Overall, while anti-CD20 therapies remain effective for RMS, their long-term use is associated with hypogammaglobulinemia and infection risk. CAR T-cell therapy offers a mechanistically distinct approach with the potential for deeper, compartment-spanning B-cell modulation, but robust evidence, especially regarding durability, long-term safety, and comparative effectiveness, is still emerging [26–28].

### CAR T-cell therapy: mechanism and lessons from hematologic and rheumatologic applications

CAR T-cells are an innovative approach to immunotherapy, distinctly different in function from endogenous T-cells. While endogenous T-cells rely on antigen presentation through the Major Histocompatibility Complex (MHC) or Human Leukocyte Antigen (HLA) molecules, CAR T-cells are engineered to bypass this restriction by directly recognizing surface antigens on target cells [29–32]feature allows them to effectively target tumor cells that evade immune detection by downregulating MHC expression. Once engaged with their antigen, CAR T-cells can directly exert cytotoxic effects without requiring activation by antigen-presenting cells (APCs) [33–36].

Structurally, CARs are designed to optimize T-cell activation, survival, and proliferation. They consist of several domains, including an extracellular antigen-binding region typically formed by a single-chain variable fragment (scFv) derived from an antibody, which mediates MHC-independent antigen recognition. This design contrasts with the native T-cell receptor (TCR), which is strictly dependent on MHC/HLA-mediated antigen presentation [37]. The hinge region provides flexibility for optimal antigen access, while the transmembrane domain anchors and stabilizes the receptor on the T-cell surface. Intracellularly, co-stimulatory and signaling domains cooperate to initiate and amplify activation signals: the co-stimulatory domain promotes T-cell survival and expansion, and the signaling domain drives cytotoxic function [29, 38, 39].

Currently, several CAR T-cell products have received regulatory approval by the United States Food and Drug Administration (FDA), the European Medicines Agency (EMA), and the United Kingdom Medicines and Healthcare products Regulatory Agency (MHRA). CD19-directed products such as tisagenlecleucel (Kymriah), axicabtagene ciloleucel (Yescarta), brexucabtagene autoleucel (Tecartus), and lisocabtagene maraleucel (Breyanzi) are indicated for B-cell malignancies including acute lymphoblastic leukemia (ALL), non-Hodgkin lymphoma (NHL), and mantle-cell lymphoma. More recently, BCMA-directed therapies, idecabtagene vicleucel (Abecma) and ciltacabtagene autoleucel (Carvykti), have been approved for relapsed or refractory multiple myeloma [40].

The CAR T-cell mechanism of action unfolds through a series of interactions with the target cell, leading to the target cell’s elimination. CAR T-cells recognize and bind to antigens on the target cell’s surface through their engineered receptors, triggering T-cell activation and cytokine secretion, which stimulates other immune cells. Activated CAR T-cells release cytotoxic molecules like granzyme and perforin, which penetrate the target cell’s membrane, inducing apoptosis. Concurrently, CAR T-cells recruit additional immune cells, such as monocytes and macrophages, augmenting the immune response against the tumor. However, this robust immune response may also contribute to Cytokine Release Syndrome (CRS), a common side effect associated with increased toxicity in patients [35, 39, 41, 42].

A unique feature of CAR T-cell therapy is its ability to persist and expand in the patient’s body after administration. Lymphodepleting chemotherapy is typically given beforehand to create a favorable environment for CAR T-cell expansion. The resulting lymphopenia promotes homeostatic proliferation and the development of memory-like CAR T cells, which can survive for extended periods and continue to recognize and eliminate cells expressing the target antigen, earning them the moniker of a “living drug” [43].

**Fig. 1:**
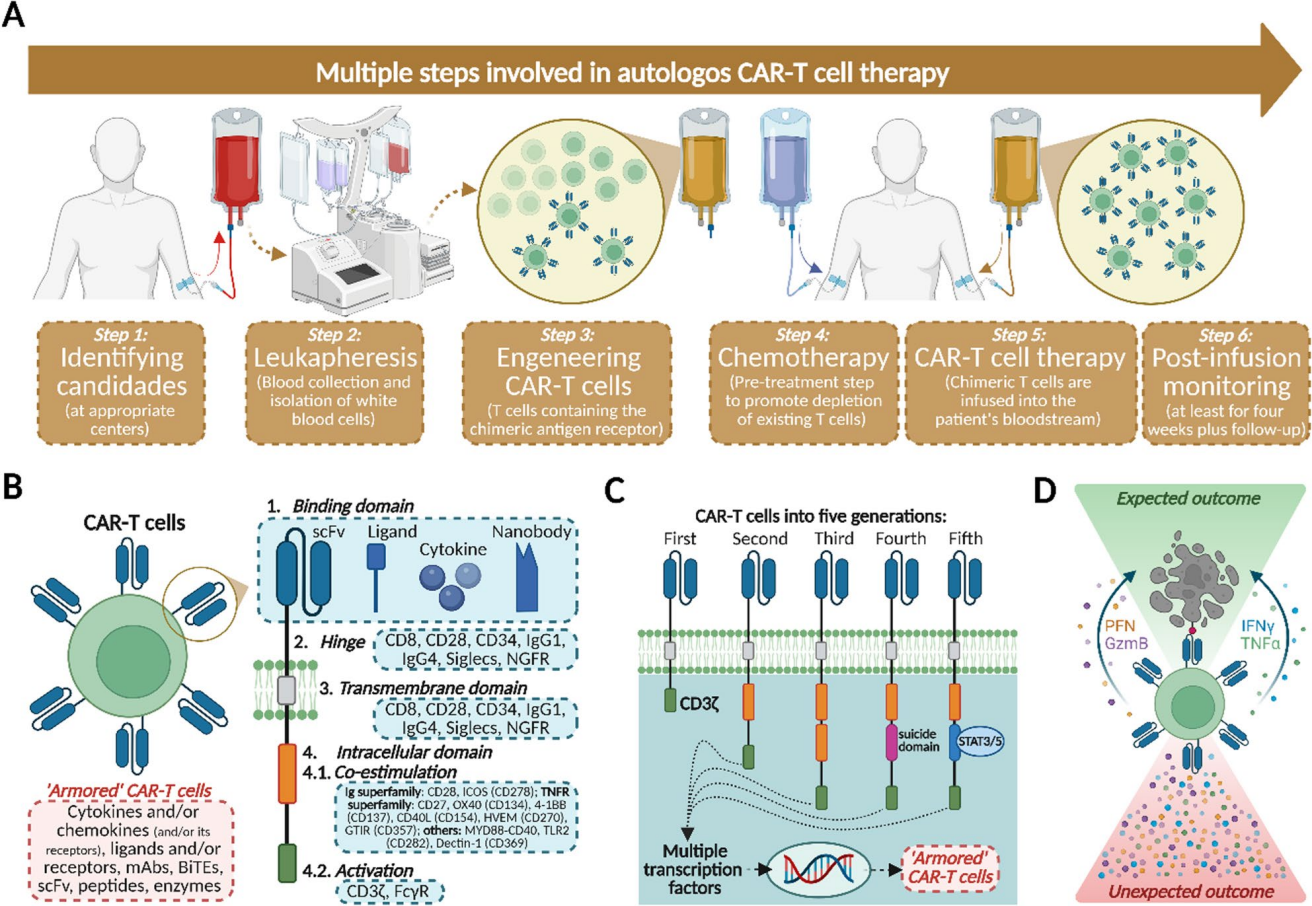
Steps for Using and Properties of CAR-T Cells. **A** Multiple steps involved in autologous CAR-T cell therapy. After selecting candidates for treatment in specialized centers (Step 1), peripheral blood is collected and white blood cells are obtained through leukapheresis (Step 2). Later, T cells are purified from other leukocytes using anti-CD3/CD28-coated beads. Purified T cells are activated, and the genetic material encoding chimeric antigen receptors (CAR) is introduced into these cells [several methods such as mRNA transfection, viral vectors, or sleeping beauty (SB) transposons can be used] (not shown). Finally, after being obtained from the donor and engineered, the autologous CAR-T cells (Step 3) are expanded before being infused into patients. Before infusion, patients receive chemotherapy (Step 4) to promote lymphocyte depletion and enhance the effectiveness of the therapy. After the infusion (Step 5), patients are monitored for at least 4 weeks, followed by a more extended period to assess the efficacy and safety of the therapy (Step 6). **B** The structure of CAR-T cells is composed by the binding domain [a single-chain fragment variant (scFV) comprising of heavy and variable light chain regions composed of an antigen-specific immunoglobin)], hinge (responsible for the transmission of receptor-binding signals), transmembrane domain (hydrophobic alpha helix that extends in the cell membrane and sustains receptor stability and surface expression), and intracellular domain (co-estimulatory and activation sites), which upon stimulation, clusters and undergoes conformational changes, thus enabling the recruitment and phosphorylation of downstream signaling proteins. **C** Variations in the intracellular signaling domains have received the most attention in optimizing CAR-T cells and allowed their categorization, so far, in five different types of engeneered cells, as follows: first generation [containing just a CD3 zeta (ζ) chain], second generation [containing a CD3ζ in addition to one co-stimulatory domain, obtained from co-stimulatory molecules such as CD28 and 4-1BB (CD137) connected to an activator domain (CD3ζ/γ chain of Fc receptor)], the third (containing both CD28 and CD137 co-stimulatory molecules in a paired fashion), the fourth (containing these as well as cytokines, chemokine receptors, and a suicide gene) – this generation, in particular, was engineered to promote cytokine-mediated killing (TRUCKs) through the release of cytokines as interleukin 12 (IL-12), IL-15 and IL-18 in the tumor tissue to overcome tumor microenvironment immunosuppression. These cytokines, to a varying degree, work synergistically promote the induction of interferon-γ (IFN-γ), tumor necrosis factor-α (TNF-α), perforin (PFN), and granzymes (as granzyme-B, GzmB) in T cells and NK cells, thus inhibiting Treg proliferation and potentially correlating with improved therapeutic effectiveness and better outcomes during antitumor immune responses. And, finally, a fifth generation (comprising an extra intracellular domain of cytokine receptors in comparison to their predecessors with a motif to bind transcription factors such as STAT3/5, allowing not only the activation of T cells and the development of memory cells, but also the stimulation of the immune system). **D** CAR T-cells display immunological characteristics similar to activated T cells. Expected outcomes include the elimination of target cells through the release of cytotoxic granules (PFN, GzmB) and cytokines (e.g., IFN-γ and TNF-α, among others), while sustaining a broader immune response within the patient. In some patients, adverse events due to the infusion of CAR-T cells may include fever, febrile neutropenia, encephalopathy, hypoxia, and hypotension. Moreover, severe adverse events, including neurotoxicity, serious infections, prolonged cytopenia, and cytokine release syndrome (CRS) may be part of unexpected outcomes of this therapy [46, 47]. Created with Biorender

CAR-T cell treatment can be either with allogenic or autologous cells. The journey of a patient through autologous CAR T-cell therapy involves a multi-step process, beginning with patient identification and ending with long-term monitoring. Step 1 identifies appropriate candidates at specialized treatment facilities. Step 2 involves leukapheresis, where white blood cells are collected from the patient through a catheter and sent to a manufacturing facility. In Step 3, the CAR T-cell product is engineered by incorporating the chimeric antigen receptor into the patient’s T-cells. Step 4 includes preparing the patient with lymphodepletion, a pre-treatment step that reduces existing T-cells to enhance CAR T-cell expansion and persistence. The most widely used lymphodepletion regimen consists of fludarabine (30 mg/m²/day for 3 consecutive days) combined with cyclophosphamide (500 mg/m²/day for 3 consecutive days), typically administered intravenously 5–7 days before CAR T-cell infusion. This combination provides transient, profound lymphocyte depletion, optimizes the engraftment of CAR T-cells, and reduces competition from endogenous lymphocytes. Step 5 is the treatment phase, where the CAR T-cell product is infused into the patient. Finally, in Step 6, patients undergo close monitoring for at least four weeks post-infusion to manage adverse events, followed by long-term follow-up [38, 44, 45] (Fig. 1A).

CAR T-cell therapy is distinct from stem cell transplantation (SCT) in both approach and mechanism (Fig. 1B-C). Unlike SCT, which uses stem cells, CAR T-cell therapy leverages genetically modified T-cells. SCT typically requires myeloablative conditioning with high-dose chemotherapy to destroy malignant and some healthy cells, necessitating the infusion of healthy cells afterward. In contrast, CAR T-cell therapy uses lower-dose lymphodepleting chemotherapy to enhance CAR T-cell persistence and expansion. SCT employs autologous stem cells (from the patient) or allogeneic stem cells (from a donor) post-conditioning. Following infusion in CAR T-cell therapy, the modified T-cells proliferate and actively engage target antigens to destroy malignant cells. Additionally, CAR T-cell therapy is more suitable for older patients, who may not be candidates for SCT due to graft-versus-host disease (GVHD) risks and decreased treatment tolerance.

Clinical trials evaluating CD19 CAR T-cell therapies in hematologic malignancies, such as large B-cell lymphoma, and in preliminary autoimmune disease studies, reveal notable efficacy. In large B-cell lymphoma, the TRANSFORM trial showed a 73% overall response rate (ORR) and 53% complete response (CR) with lisocabtagene maraleucel (liso-cel) [48]. ZUMA-1 with axicabtagene ciloleucel (axi-cel) had an ORR of 83% and CR rate of 59% [49], while JULIET with tisagenlecleucel (tisa-cel) had an ORR of 53% and a CR of 39% [50]. In autoimmune conditions like SLE and lupus nephritis (LN), most patients achieved disease activity-free status and could discontinue immunosuppressants [27, 51–55]. Similar results were observed in inflammatory myopathies (IIM) and systemic sclerosis (SSc), where patients maintained low reliance on immunosuppressants [56–61]. These findings remain preliminary, and CAR T-cell treatments for autoimmune diseases are not yet approved by FDA, EMA and MHRA [27, 49, 59, 62–64].

Regarding the safety of CAR-T cell treatment, in the acute post-infusion phase, two main adverse events can occur: cytokine release syndrome (CRS) and immune effector cell-associated neurotoxicity syndrome (ICANS). CRS is a common and serious side effect of CAR T-cell therapy, occurring after CAR T-cells bind to target cells (Fig. 1D). Upon activation, CAR T-cells proliferate and release pro-inflammatory cytokines, which stimulate macrophages and dendritic cells, creating a cytokine cascade that amplifies the inflammatory response. In parallel, the lysis of target cells releases cellular debris and damage-associated molecular patterns (DAMPs) that further amplify cytokine production. CRS symptoms range from mild to severe, including fever, myalgia, nausea, transient hypoxia, and hypotension. In severe cases, multiple organ dysfunction syndrome (MODS) may occur [65]. CRS occurs in 30–100% of hematology patients treated with CAR T therapy and has also been observed in autoimmune disease trials, where mild forms of CRS are more common. CRS requires vigilant monitoring and intervention to mitigate risks and maintain patient safety [27, 41, 42, 66–69].

ICANS is another potential side effect, resulting from cytokine-mediated inflammation within the brain. Elevated cytokine levels disrupt the blood-brain barrier, allowing pro-inflammatory molecules to infiltrate and activate neurons and microglia. Symptoms include aphasia, delirium, tremor, and, in severe cases, seizures and respiratory depression. ICANS occurs in 20–60% of lymphoma patients treated with CAR T-cell therapy. Although less common in autoimmune applications of CAR T-cell therapy, ICANS is a serious adverse event, necessitating management to ensure symptoms are usually transient and resolve with appropriate intervention [27, 37, 41, 68–72].

Two primary factors influence the severity of CRS and ICANS: the type of co-stimulatory domain used in the CAR T-cell construct and the patient’s target or lesion burden. Different co-stimulatory domains confer varying risks for CRS and ICANS, with CAR T-cells containing a CD137 domain associated with lower rates and grades of these toxicities compared to constructs with other domains. High disease burden, such as in leukemia or multiple myeloma, also correlates with increased risk and severity of CRS and ICANS, suggesting that disease extent may exacerbate the inflammatory response during CAR T-cell therapy [73–75]. In cases of high B-cell burden, such as certain hematological malignancies, the abundant target antigen presence leads to increased CAR T-cell activation, heightening the risk of severe side effects like CRS and ICANS [76, 77]. In large B-cell lymphoma (LBCL) trials, CRS and ICANS rates vary depending on the construct. In TRANSCEND (lisocabtagene maraleucel (liso-cel)), 42% of patients experienced CRS (2% severe) and 30% had neurologic events (10% severe) [62]. ZUMA-1, axicabtagene ciloleucel (axi-cel), showed higher rates: 93% CRS and 64% neurologic events [49]. JULIET, tisagenlecleucel (tisa-cel) had intermediate rates, with 57% CRS and 20% neurologic events [49, 62, 63, 78–80].

Similarly, BCMA-directed CAR T therapies in hematologic settings report high toxicity. In KarMMa (idecabtagene vicleucel (ide-cel)), 84% of multiple myeloma patients had CRS (5% ≥ grade 3), and 18% had neurologic events (3% severe) [81]. In CARTITUDE-1 (ciltacabtagene autoleucel (cilta-cel)), 95% had CRS (4% severe) and 21% had neurologic events (9% severe) [82]. These findings highlight the importance of CAR construct design, disease burden, and patient-specific factors in toxicity outcomes.

Beyond CRS and ICANS, several additional toxicities have been described in hematologic CAR T-cell studies. Immune effector cell–associated hemophagocytic syndrome (IEC-HS/ICAHT) is a rare but serious hyperinflammatory condition, typically arising later in the course and characterized by elevated ferritin, cytopenias, and organ dysfunction. Hypogammaglobulinemia and related infection risks are frequent immune consequences of sustained B-cell depletion and lymphodepletion, often necessitating immunoglobulin replacement and anti-infective prophylaxis. These events highlight the need for individualized monitoring and supportive care measures, similar to those used in anti-CD20 therapy recipients [27, 64, 83, 84].

Furthermore, isolated reports from hematologic cohorts have described delayed neurological syndromes following BCMA-directed CAR T-cell therapy, including parkinsonism, cognitive impairment, and Guillain–Barré–like presentations occurring weeks to months post-infusion. While such events remain exceedingly rare and causality remains uncertain, they highlight the need for vigilant long-term neurological surveillance and standardized neurotoxicity reporting in ongoing and future studies [84].

In the medium-long term after CAR-T cell therapy, continuous monitoring for toxicity remains essential [35, 36, 38, 39, 44, 85]. The FDA and EMA have been investigating the risk of secondary malignancies, specifically T-cell malignancies, in lymphoma patients treated with CAR T-cell therapy. Studies suggest that the risk of secondary malignancies may be five times higher in these patients compared to the general population. The FDA’s FAERS database reported a T-cell malignancy rate of 0.275%, with 22 cases out of 8,000 CAR T-cell-treated patients [86]. At the University of Pennsylvania, a rate of 0.2% was observed (1 in 449 patients), including CAR-negative malignancies [87]. In June 2024, the EMA reviewed 38 reported cases of secondary T-cell malignancies following BCMA- or CD19-directed CAR T-cell therapy to clarify the underlying risk factors. lthough these investigations are ongoing, the contribution of factors such asage, prior therapies, and immune status remain uncertain, reinforcing the recommendation for lifelong monitoring in CAR T-cell recipients to enable early detection and management potential malignancies [88–94].

In summary, the spectrum of toxicities associated with CAR T-cell therapy extends across acute, subacute, and delayed phases, involving multiple organ systems. In the acute post-infusion period, CRS and ICANS remain the predominant complications, reflecting cytokine-mediated systemic and neurological inflammation. Subacute events, such as IEC-HS/ICAHT, cytopenias, and infections, typically emerge within the first weeks after treatment and require close hematologic and metabolic monitoring. In the delayed phase, rare but clinically important events, including neurodegenerative-like syndromes, secondary T-cell malignancies, and hypogammaglobulinemia, underscore the need for long-term surveillance and individualized follow-up.

## Materials and methods

Not applicable.

## Results

Not applicable. 

### Discussion: cell therapy in neurological diseases: current understandings and future potential

In neuroimmune disorders, self-antigens can initiate a self-perpetuating cycle of immune cell activation, autoantibody production, chronic inflammation, and tissue damage, forming an autoimmune loop. This loop may begin with tissue damage caused by infections, physical trauma, chemical stressors or unknown triggers, which leads to autoantibody production. B cells undergo affinity maturation, differentiating into plasmablasts (PB) and plasma cells (PC), which generate autoantibodies. Once initiated, the autoimmune cascade is sustained by a complex interaction between dendritic cells (DCs), B cells, and T-cells, establishing an “autoimmune enhancer loop” [95]. This process can either be limited, allowing for inflammation resolution and tissue repair, or become chronic and persistent, contributing to autoimmunity and chronic tissue inflammation [64]. In this context, CAR T-cell therapy, through their targeted and long-lasting therapeutic approach, may represent a valid strategy to break the pathologic autoimmune loop.

As CAR T-cell therapy expands into neuroimmune indications, early clinical observations suggest that toxicity profiles are generally milder and more transient than in hematologic malignancies, likely reflecting the lower antigen burden and more regulated immune activation within the CNS and peripheral compartments.

#### Multiple sclerosis

In MS, preclinical and early clinical data suggest that CAR-T cell therapy may address both acute inflammatory flares and smoldering CNS pathology, positioning CAR T-cells as a transformative modality under active investigation. CD19 CAR-T therapy shows efficacy in MS across preclinical and clinical settings. In a mouse model, treatment reduced clinical scores and neuroinflammation within 28 days [96]. However, it is noteworthy that part of this therapeutic effect was also observed in lymphodepleted control mice treated with cyclophosphamide alone, suggesting that the conditioning regimen itself may exert independent immunomodulatory effects [97], such as transient depletion of autoreactive lymphocytes and altered cytokine milieu, contributing to clinical improvement. These findings highlight the need to disentangle the relative contributions of lymphodepletion and CAR T-cell activity in preclinical and early human studies.

Translating these preclinical observations to the clinical setting, initial case reports in PMS have demonstrated similar immune reprogramming and sustained B-cell depletion following CD19 CAR T-cell therapy. In two individual progressive MS patients (one SPMS, one PPMS) treated compassionately with fully human CD19 CAR T cells (KYV-101), CAR T-cell expansion was observed in both peripheral blood and CSF, accompanied by sustained systemic B-cell depletion and stable EDSS scores at days 28 and 100. Importantly, no ICANS or other severe neurotoxicity occurred despite CAR T-cell trafficking into the CSF, and one patient exhibited a significant and sustained reduction in intrathecal IgG synthesis by day 64, suggesting CNS-compartmental effects on CD19^+^ target cells [98].

Furthermore, an ongoing phase 1 clinical trial (NCT04561557) is evaluating autologous anti-BCMA CAR T-cell therapy in progressive MS. The first report includes five patients with treatment-refractory PMS (one PPMS, four SPMS). Safety outcomes were favorable, with only grade 1 CRS observed in all participants and all grade ≥ 3 cytopenias occurring within the first 40 days post-infusion. No ICANS or other serious neurological toxicity was reported. Immunologic analyses demonstrated marked depletion of CD138 + plasma cells within CNS compartments, prolonged CAR T-cell expansion in both blood and CSF, and an effector-dominant phenotype (CD8⁺GZMB⁺/CD8⁺GZMK⁺) with reduced exhaustion markers. Additionally, CSF microglial activation was attenuated, suggesting potential modulation of compartmentalized neuroinflammation. Although these early biologic effects are encouraging, follow-up remains limited and long-term safety, durability, and clinical efficacy require confirmation in larger cohorts [99].

To note, the immunomodulatory effects of prior disease-modifying therapies in MS patients establish a baseline of immune dysregulation, raising theoretical concerns that CAR T-cell–mediated T-cell activation could precipitate excessive immunostimulation. This hyperactivation may amplify known toxicities such as CRS and ICANS. While long-term durability remains under investigation, preliminary evidence described above suggests sustained clinical benefits without emergence of delayed adverse effects.

#### Myelin oligodendrocyte glycoprotein antibody-associated disease (MOGAD)

In myelin oligodendrocyte glycoprotein antibody-associated disease (MOGAD), current therapeutic strategies face substantial limitations inherent to chronic management paradigms. Maintenance protocols typically involve immunomodulatory agents universally off-label, with sparse controlled efficacy and safety data specific to this disease [100]. Notably, B-cell–depleting agents like rituximab demonstrate only partial efficacy in this disease, with approximately 30% of patients experiencing breakthrough relapses despite achieving peripheral B-cell ablation [101]. This divergence underscores distinct pathophysiological mechanisms between antibody-mediated neuroinflammatory disorders. These challenges highlight an urgent need for mechanistically targeted therapies with durable efficacy and improved tolerability.

Emerging biologics and cellular immunotherapies are under investigation to address this unmet need. In this context, CD19-directed CAR T-cell therapy was recently used in an 18-year-old man with highly relapsing, treatment-refractory MOGAD (six optic neuritis episodes over six years). After infusion of autologous ARI-0001 CAR T-cells following conventional lymphodepletion, treatment was well tolerated with no acute complications. The patient achieved rapid and complete CD19⁺ B-cell depletion by day + 7, with B-cell reconstitution at day + 141 showing a predominantly naïve phenotype and persistently low memory B-cells and plasmablasts. MOG-IgG became undetectable shortly after infusion and remained negative throughout 12-month follow-up. Aside from a single optic neuritis episode on day + 29, no further relapses or new neurodegeneration occurred without maintenance immunotherapy [80].

Preclinical work in B-cell–dependent EAE models provides complementary mechanistic support: anti-CD19 CAR T-cells, combined with cyclophosphamide conditioning, depleted peripheral and CNS B-cells and reduced clinical and histologic disease severity. Notably, some clinical benefit also occurred in cyclophosphamide-conditioned control T-cell recipients, indicating that lymphodepletion may contribute to disease amelioration independently of CAR specificity. Together, these early findings suggest that CD19 CAR T-cell therapy may be a promising option for refractory MOGAD, while highlighting the need for controlled studies to define durability and disentangle CAR-specific versus lymphodepletion-driven effects [96].

#### Neuromyelitis optica spectrum disorder (NMOSD)

Neuromyelitis optica spectrum disorder (NMOSD) exhibits distinct pathobiological trajectories compared to MS, characterized by acute inflammatory events that frequently result in incomplete neurological recovery. Notably, up to 60% of NMOSD patients experience recurrent relapses within 12 months of disease onset, underscoring the critical need for prompt immunomodulatory intervention to mitigate accrual of irreversible disability [102]. Emerging evidence further cautions against cessation of immunosuppressive regimens due to elevated relapse risks post-discontinuation, reinforcing current guidelines advocating for indefinite maintenance therapy. A therapeutic gap persists for refractory NMOSD subpopulations exhibiting poor response to conventional biologics, rituximab, or complement inhibitors. These individuals face disproportionate risks of life-threatening relapses, cumulative neuroaxonal injury, and premature mortality.

In this context, BCMA-directed CAR T-cell therapy has emerged as a promising strategy for patients with relapsing or treatment-refractory AQP4-IgG–positive NMOSD. In a phase 1, single-arm trial (NCT04561557), 12 patients (median age 49.5 years; 83% women) received autologous BCMA CAR T cells. CRS occurred in all individuals but was limited to grade 1–2; the most frequent grade ≥ 3 events were transient hematologic toxicities. During a median follow-up of 5.5 months, 11 of 12 patients experienced no relapses, most reported improved disability and quality of life, and serum AQP4-IgG titers showed a downward trend. CAR T-cell expansion correlated with clinical responses, and persistence beyond six months was observed in 17% of patients [103].

Mechanistic single-cell multi-omics analyses of paired CSF and blood samples demonstrated that proliferating cytotoxic-like CD8⁺ CAR T-cell clones act as key effectors in autoimmunity. Anti-BCMA CAR T cells efficiently crossed the blood–CSF barrier, eliminated plasmablasts and plasma cells within the CSF, suppressed neuroinflammatory signaling, and promoted microglial quiescence. CAR T cells exhibited chemotaxis-enhancing features and a CD44-positive early memory phenotype associated with persistence in autoimmune settings; importantly, they showed reduced cytotoxicity signatures compared with CAR T cells used in hematologic malignancies, consistent with the milder toxicity profile observed in NMOSD [104].

#### Myasthenia Gravis (MG) and Lambert-Eaton myasthenic syndrome (LEMS)

MG has also emerged as a key target for CAR T-cell innovation, particularly through RNA-based platforms designed to improve safety. The MG-001 trial evaluated Descartes-08, an autologous anti-BCMA RNA CAR T-cell product administered without lymphodepleting chemotherapy, in 14 adults with generalized MG. Across phase 1b/2a cohorts, Descartes-08 showed a favorable acute safety profile with no CRS, no ICANS, and no dose-limiting toxicities. The most common adverse events, headache, nausea, vomiting, and transient low-grade fever, resolved within 24 h and were not associated with cytokine-release biomarkers. Clinically, patients demonstrated rapid and sustained improvement: at 12 weeks, MG-ADL decreased by a mean of 6 points, QMG by 7 points, MGC by 14 points, and MG-QoL15r by 9 points, with benefits persisting up to 9 months of follow-up [77, 104–106].

Beyond MG, early evidence also supports CAR T-cell efficacy in Lambert–Eaton myasthenic syndrome (LEMS). A recent case report described treatment of a patient with isolated idiopathic LEMS using autologous anti-CD19 CAR T cells, leading to robust CAR T expansion, predominantly CD4⁺ TEMRA-like cells, and subsequent B-cell depletion. VGCC antibody titers declined markedly, accompanied by significant clinical improvement including an eight-fold increase in walking distance. Toxicity was manageable, limited to grade 2 CRS and intermittent neutropenia, suggesting that anti-CD19 CAR T-cell therapy may represent a therapeutic option for severe, treatment-refractory LEMS [107, 108].

#### Idiopathic inflammatory myopathies (IIM)

In Idiopathic Inflammatory Myopathies (IIM) (myositis), the investigational CD19-directed CAR T-cell product CABA-201 is being assessed in a Phase 1/2 open-label trial (NCT 06154252, RESET-Myositis), representing one of the first applications of CAR T therapy beyond classic neuroimmune disorders.

#### Stiff-person syndrome (SPS) and anti-GAD disease

Evidence for CAR T-cell therapy in stiff-person syndrome (SPS) comes from case reports. In the first published case, a 69-year-old woman with 9-year refractory SPS received autologous KYV-101 and showed marked reduction in leg stiffness, > 100% increase in walking speed, and improvement in daily walking distance from < 50 m to > 6 km within 3 months, with a 40% reduction in benzodiazepines and only low-grade CRS. A second compassionate-use case involved a 62-year-old woman with 14-year anti-GAD SPS and comorbid MG, nearly non-ambulatory before treatment; by month 6 she improved to 500 m walking distance, with anti-GAD titers falling from 1:320 to 1:32, and experienced only grade 2 CRS and moderate leukopenia without serious infections. Manufacturing data from 20 autoimmune patients (13 neurological) demonstrated high viability, robust expansion, and consistent CD19-dependent activity of KYV-101, supporting feasibility across indications. Altogether, these early reports indicate clinically meaningful benefit with manageable safety, though controlled trials are required to confirm efficacy and durability in SPS [79, 109, 110].

A key question in the clinical development of CAR T-cell therapy for autoimmune neurological diseases is whether this approach acts as a single “immune reset” or requires periodic maintenance. Early clinical data from autoimmune indications such as SLE and MG suggest that a single infusion can induce durable remission, even after peripheral B-cell reconstitution, indicating a potential long-term rebalancing of immune tolerance [27, 54, 64]. This concept is supported by observations of deep B-cell lineage depletion and prolonged molecular quiescence of autoreactive clones following CAR T-cell therapy. However, the persistence and durability of this immune reset likely differ across diseases and depend on antigen specificity, CAR T-cell persistence, and the degree of compartmentalized inflammation within the CNS. In disorders such as MS and NMOSD, relapse risk may re-emerge once B-cell lineages repopulate, potentially necessitating repeat dosing or combination with adjunct immunotherapies [27, 64]. Defining the duration and mechanisms of this immune reset will therefore be critical for optimizing CAR T-cell treatment schedules and long-term management strategies.

While early clinical data indicate encouraging safety outcomes in autoimmune and neurological applications, potential on-target, off-tissue effects require continued evaluation. Notably, Parker et al. (Cell, 2020) identified CD19 expression in brain mural cells, pericytes and vascular smooth muscle cells that support BBB integrity, using single-cell transcriptomic and protein-level analyses. Although no direct neurovascular toxicity has been reported in autoimmune or hematologic CAR T trials to date, this finding suggests a potential on-target mechanism for neurotoxicity and underscores the importance of continued neurological monitoring to clarify long-term CAR T-cell safety [111].

Importantly, the long-term consequences of persistent CAR T-cell engraftment in the CNS remain unknown. Prolonged CAR T-cell activity could theoretically contribute to chronic low-grade neuroinflammation or subtle BBB alterations, even in the absence of overt early toxicity. Given the limited follow-up available in current autoimmune and neurological cohorts, safety conclusions remain provisional, and long-term clinical, imaging, and biomarker surveillance will be essential to fully characterize delayed risks.

Although such events have been rare and causality uncertain, delayed neurological syndromes, including parkinsonism, cognitive impairment, and Guillain–Barré–like presentations, have been reported following BCMA-directed CAR T-cell therapy, reinforcing the importance of long-term neurocognitive and motor surveillance in CAR T-treated patients [84]. In parallel, systemic complications such as IEC-HS/ICAHT, hypogammaglobulinemia, and infection risk should remain part of comprehensive post-treatment surveillance to ensure early recognition and management of delayed immune toxicities [74, 112, 113].

These findings emphasize the therapeutic versatility of CAR T-cell therapy across neurologic autoimmune disorders. In general, safety profiles are notably milder than in hematologic settings, and early results point toward durable disease control. Indeed, data on the use of CD19- and BCMA-directed CAR T-cell therapies for autoimmune and neurological indications shows better safety profiles. In MS, 50% of treated subjects had CRS, none exceeding grade 2, and no ICANS reported [98]. MG, Lambert-Eaton myasthenic syndrome (LEMS), stiff person syndrome (SPS), and MOGAD showed 60% CRS and 20% mild ICANS [78, 106]. In NMOSD, BCMA-directed therapy led to 100% CRS, all mild. In MG, no CRS or ICANS were reported [104, 114, 115].

A cohort of 28 patients with neurologic diseases receiving CAR T therapy showed promising safety: 58% had low-grade CRS, only one mild ICANS case, and no severe infections. Patients with MS, NMOSD, MG, SPS, and MOGAD showed reduced disease activity and could taper immunotherapies [98, 116].

A case in MG illustrates effective side-effect management. Following infusion, peak CAR T expansion occurred on day 9 with low-grade CRS and ICANS. Corticosteroids were started on day 5, along with anti-IL-6 and anti-IL-1. These were tapered by day 15, and the patient was discharged by day 22. This case highlights how timely, targeted interventions guided by close monitoring can mitigate CAR T toxicity in autoimmune settings [78, 104, 106, 117].

Lower B-cell burden in autoimmune diseases may partly explain the reduced toxicity. In contrast to hematologic malignancies with high antigen load and more intense T-cell activation, autoimmune conditions may induce weaker CAR T activation, resulting in milder CRS and ICANS [61, 77, 106].

In conclusion, personalized strategies, tailored by disease context and antigen burden, will be key to maximizing benefit and minimizing risk [64]. A collaborative care model involving neurologists and hematologists is recommended to optimize CAR T-cell therapy outcomes, particularly for managing acute and long-term toxicities. This partnership encompasses various CAR T-cell treatment phases, from patient eligibility assessment, including leukapheresis and lymphodepleting chemotherapy, to managing CAR T-cell infusion and monitoring toxicities such as CRS and ICANS. Post-treatment, this model facilitates long-term disease management and neurological monitoring, ensuring comprehensive patient care and optimizing therapeutic efficacy while addressing potential neurological complications. This interdisciplinary approach highlights the importance of combined expertise in managing CAR T-cell therapy complexities [64, 118] (Table 1). 

**Table 1:** Ongoing and planned CAR T-cell clinical trials targeting autoimmune neurological diseases

Disease	NCT no.	Antigen	Phase	Status	T cell source
MS	NCT04561557	BCMA	Phase 1	Ongoing	Autologous
MS	NCT06138132	CD19	Phase 1	Active	Autologous
MS	NCT06220201	CD19	Phase 1	Recruiting	Autologous
MS	NCT06384976	CD19	Phase 2	Not yet recruiting	Autologous
MG	NCT06193889	CD19	Phase 2	Recruiting	Autologous
MG	NCT05828225	CD19	Phase 1	Recruiting	Autologous
MG	NCT05451212	MuSK	Phase 1	Recruiting	Autologous
MG	NCT04146051	BCMA	Phase 1/2	Recruiting	Autologous
IIM	NCT06154252	CD19	Phase 1/2	Recruiting	Autologous
IIM	NCT06298019	CD19	Phase 1	Not yet recruiting	Autologous
NMOSD	NCT05828212	CD19	Phase 1	Recruiting	Autologous

*CAR* Chimeric Antigen Receptor, *T cell* T lymphocyte, *MS* Multiple Sclerosis, *MG* Myasthenia Gravis, *IIM* Idiopathic Inflammatory Myopathies, *NMOSD* Neuromyelitis Optica Spectrum Disorder, *CD19* Cluster of Differentiation 19, *MuSK* Muscle-Specific Kinase, *BCMA* B Cell Maturation Antigen, *NCT* National Clinical Trial, *Phase 1/Phase 2* Clinical trial phases assessing safety and efficacy, *Autologous* Derived from the same individual

## Conclusions

CAR T-cell therapy represents a transformative advancement in the treating of multiple sclerosis and related neuroinflammatory disorders. Unlike traditional immunosuppressive strategies, which often require lifelong administration and provide only partial control, CD19- and BCMA-directed CAR T cells offer the potential for deep immunologic reset and sustained drug-free remission. Preliminary clinical results in MS, MOGAD, NMOSD, and MG demonstrate promising efficacy and manageable safety profiles, with low rates of high-grade CRS or ICANS. These findings, combined with the capacity of CAR T cells to eliminate tissue-resident autoreactive B cells, something conventional therapies fail to achieve, suggest a path toward long-term disease control or even cure. As ongoing trials expand our understanding, CAR T-cell therapy is poised to redefine the therapeutic landscape of autoimmune CNS disorders, shifting from chronic suppression to targeted immune reprogramming.

## Data Availability

Not applicable as it is a narrative review article.
